# Perineal wound closure using gluteal turnover flap or primary closure after abdominoperineal resection for rectal cancer: study protocol of a randomised controlled multicentre trial (BIOPEX-2 study)

**DOI:** 10.1186/s12893-020-00823-7

**Published:** 2020-07-23

**Authors:** Sarah Sharabiany, Robin D. Blok, Oren Lapid, Roel Hompes, Wilhelmus A. Bemelman, Victor P. Alberts, Bas Lamme, Jan H. Wijsman, Jurriaan B. Tuynman, Arend G. J. Aalbers, Geerard L. Beets, Hans F. J. Fabry, Ivan M. Cherepanin, Fatih Polat, Jacobus W. A. Burger, Harm J. T. Rutten, Robert J. I. Bosker, Koen Talsma, Joost Rothbarth, Cees Verhoef, Anthony W. H. van de Ven, Jarmila D. W. van der Bilt, Eelco J. R. de Graaf, Pascal G. Doornebosch, Jeroen W. A. Leijtens, Jeroen Heemskerk, Baljit Singh, Sanjay Chaudhri, Michael F. Gerhards, Tom M. Karsten, Johannes H. W. de Wilt, Andre J. A. Bremers, Ronald J. C. L. M. Vuylsteke, Gijsbert Heuff, Anna A. W. van Geloven, Pieter J. Tanis, Gijsbert D. Musters

**Affiliations:** 1 Department of Surgery, Amsterdam UMC, Cancer Centre Amsterdam, University of Amsterdam, Amsterdam, The Netherlands; 2 LEXOR, Centre for Experimental and Molecular Medicine, Oncode Institute, Cancer Centre Amsterdam, Amsterdam UMC, University of Amsterdam, Amsterdam, The Netherlands; 3 Department of Plastic Surgery, Amsterdam UMC, University of Amsterdam, Amsterdam, The Netherlands; 4 Department of Surgery, Albert Schweitzer Hospital, Dordrecht, the Netherlands; 5 Department of Surgery, Amphia Hospital, Breda, The Netherlands; 6 Department of Surgery, Amsterdam UMC, Cancer Centre Amsterdam, Free University, Amsterdam, The Netherlands; 7 Department of Surgery, Antoni van Leeuwenhoek Hospital-Netherlands Cancer Institute, Amsterdam, The Netherlands; 8 Department of Surgery, Bravis Hospital, Roosendaal, The Netherlands; 9 Department of Surgery, Canisius Wilhelmina Hospital, Nijmegen, The Netherlands; 10 Department of Surgery, Catharina Hospital, Eindhoven, The Netherlands; 11 GROW School of Oncology and Developmental Biology, University of Maastricht, Maastricht, The Netherlands; 12 Department of Surgery, Deventer Hospital, Deventer, The Netherlands; 13 Department of Surgery, Erasmus Medical Centre, Rotterdam, The Netherlands; 14 Department of Surgery, Flevo Hospital, Almere, The Netherlands; 15 Department of Surgery, IJsselland Hospital, Capelle aan den Ijssel, The Netherlands; 16 Department of Surgery, Laurentius Hospital, Roermond, The Netherlands; 17 Department of Surgery, Leicester Hospital, Leicester, UK; 18 Department of Surgery, OLVG Hospital, Amsterdam, The Netherlands; 19 Department of Surgery, Radboud University Medical Centre, Nijmegen, The Netherlands; 20 Department of Surgery, Spaarne Gasthuis, Haarlem, The Netherlands; 21 Department of Surgery, Tergooi Hospital, Hilversum, The Netherlands

**Keywords:** Abdominoperineal resection, Rectal cancer, Primary perineal wound closure, Gluteal turnover flap, Perineal wound infection and perineal wound healing

## Abstract

**Background:**

Abdominoperineal resection (APR) for rectal cancer is associated with high morbidity of the perineal wound, and controversy exists about the optimal closure technique. Primary perineal wound closure is still the standard of care in the Netherlands. Biological mesh closure did not improve wound healing in our previous randomised controlled trial (BIOPEX-study). It is suggested, based on meta-analysis of cohort studies, that filling of the perineal defect with well-vascularised tissue improves perineal wound healing. A gluteal turnover flap seems to be a promising method for this purpose, and with the advantage of not having a donor site scar. The aim of this study is to investigate whether a gluteal turnover flap improves the uncomplicated perineal wound healing after APR for rectal cancer.

**Methods:**

Patients with primary or recurrent rectal cancer who are planned for APR will be considered eligible in this multicentre randomised controlled trial. Exclusion criteria are total exenteration, sacral resection above S4/S5, intersphincteric APR, biological mesh closure of the pelvic floor, collagen disorders, and severe systemic diseases. A total of 160 patients will be randomised between gluteal turnover flap (experimental arm) and primary closure (control arm). The total follow-up duration is 12 months, and outcome assessors and patients will be blinded for type of perineal wound closure. The primary outcome is the percentage of uncomplicated perineal wound healing on day 30, defined as a Southampton wound score of less than two. Secondary outcomes include time to perineal wound closure, incidence of perineal hernia, the number, duration and nature of the complications, re-interventions, quality of life and urogenital function.

**Discussion:**

The uncomplicated perineal wound healing rate is expected to increase from 65 to 85% by using the gluteal turnover flap. With proven effectiveness, a quick implementation of this relatively simple surgical technique is expected to take place.

**Trial registration:**

The trial was retrospectively registered at Clinicaltrials.gov NCT04004650 on July 2, 2019.

## Background

Historically, abdominoperineal resection (APR) has been the standard treatment for (low) rectal cancer. Although most rectal cancer patients nowadays undergo a restorative total mesorectal excision (TME), an APR is still performed in selected patients with very low tumours and/or poor sphincter function. A drawback of APR is the resulting perineal defect that still poses a high risk of morbidity, especially in patients who have undergone pre-operative (chemo)radiotherapy [1]. Early perineal wound complications occur in up to 35% of patients, and 10% of patients still have a wound complication even 1 year after the operation [2, 3].

The high rate of perineal morbidity after primary wound closure has resulted in a continuous discussion on alternative closure methods for the perineal wound after APR. Options include biological mesh closure and (muscle) flap assisted closure. Biological mesh closure has been investigated in our previous randomised controlled trial (BIOPEX-study), and showed no superiority in perineal wound healing compared to primary closure. Biological mesh closure did result in a lower perineal hernia rate 1 year after APR (13% versus 27%; *P* = 0.0316) [2]. The absence of improvement in perineal wound healing after biological mesh closure is probably related to the formation of a dead space between the mesh and the perineal skin after APR. This dead space is prone to fluid accumulation, which can subsequently get infected with abscess formation.

The hypothesis is that filing of the dead space by well-vascularised soft tissue can prevent fluid accumulation, and thereby reduce infectious complications and abscess formation. Several myocutaneous and fasciocutaneous flaps have been used for this purpose. In a systematic review of cohort studies, muscle flap closure of the perineal wound showed an improvement of 20% compared to primary closure of perineal defects after oncological resection [4]. However, downsides of these flaps are the donor-site and recipient-site morbidity that are both often neglected, besides the complexity of the reconstructive procedures with the need for the presence of a plastic surgeon, and substantially increased operative time. Especially in the case of a rectus abdominis muscle flap, the flap interferes with the benefits of a laparoscopic approach of the APR and gives a high risk of donor site morbidity. Furthermore, there might be an impact on cosmesis due to an additional scar.

A gluteal turnover flap is a small transposition flap consisting of the adjacent skin and subcutaneous tissue of one of the buttocks. This flap is deepithelialised and dissected down to the gluteal muscle, it is perfused through perforating blood vessels from the underlying muscle. The flap is hinged into the resected space of the anal sphincter complex, and the dermis is stitched to the contralateral levator remnant as a pelvic floor closure. The skin is closed in layers over the flap in the midline, thereby not creating an additional scar, and minimizing donor-site morbidity. Moreover, it is a relatively simple procedure, which is easy to learn, and can be performed without the need of a plastic surgeon.

Potentially, the gluteal turnover flap both improves wound healing by filling the dead space, as well as preventing hernia formation by stitching deepithelialised dermis to the levator remnant. A pilot study of this gluteal turnover flap showed no flap necrosis, and no major morbidity requiring radiological or surgical re-intervention within 30 days [5].

The aim of this randomised controlled multicentre trial is to compare the effect of gluteal turnover flap closure of the perineal wound with primary perineal wound closure after APR for rectal cancer with regard to perineal wound healing.

## Methods/design

### Objective

The objective of this study is to investigate the effect of gluteal turnover flap closure of the perineal wound with primary closure of the perineal wound after APR for rectal cancer. The hypothesis is that gluteal turnover flap reconstruction increases the uncomplicated perineal wound healing rate, and decreases the occurrence of perineal hernia.

### Design

**Fig. 1:**
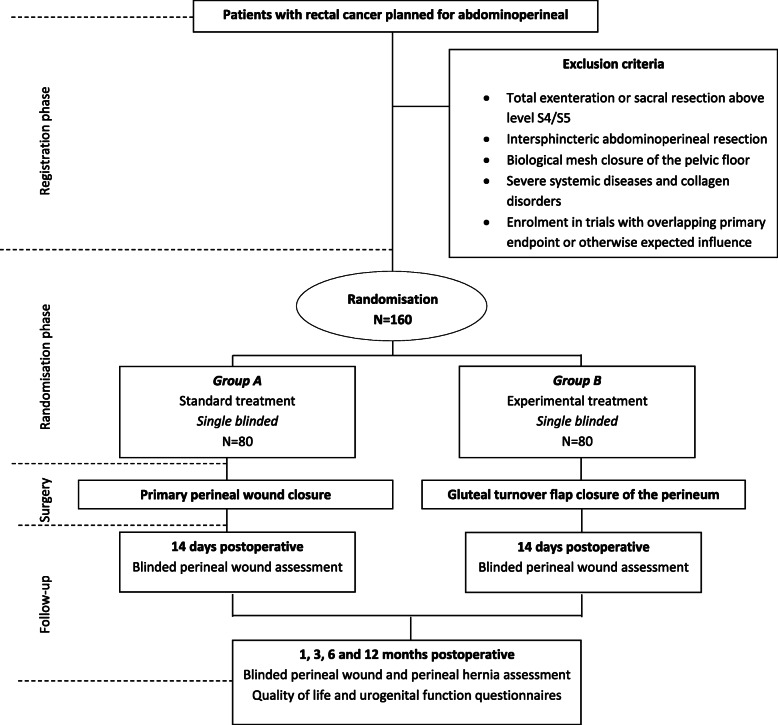
Flow-diagram: study design

In this multicentre, single-blind, randomised controlled trial, patients will be randomised between gluteal turnover flap closure (experimental arm) and primary wound closure (control arm) (Fig. 1). A block randomisation of 1:1 will be used, stratified for primary or recurrent rectal cancer and neo-adjuvant (chemo)radiotherapy. The patient is blinded to the allocation of treatment. The study will be carried out in five academic centres, 11 teaching hospitals and two non-teaching hospitals. In centres without experience in gluteal turnover flap reconstruction, the procedure will be supervised by one of the principal investigators (PJT or RH) or the plastic surgeon (OL) until a standardised and quality controlled gluteal turnover flap procedure is assured.

Perineal wound healing will be assessed at 14 days, and 1, 3, 6 and 12 months postoperatively using the Southampton wound score (Table 1). Evaluation will be performed by an independent observer not aware of treatment allocation. The perineal wound will be scored with regard to complications and hernia. In addition, a photograph of the perineal wound will be made. A CT-scan will be performed according to the follow-up, and will be assessed for the occurrence of presacral or perineal abscesses and perineal herniation. Quality of Life and urogenital questionnaires will be collected at each follow-up moment (EQ-5D, EORTC-30, EORTC-29, SF36, UDI-6, IIQ-7, IIEF, FSFI, FSDS-R). Additionally, all perineal wound events, re-admissions and re-interventions will be scored.

**Table 1:** Southampton wound scoring system

Grade	Appearance
0 – Normal healing	
1 – Normal healing with mild bruising or erythema	A – some bruising
	B – considerable bruising
	C – mild erythema
2 – Erythema plus other signs of inflammation	A – at one point
	B – around sutures
	C – along wound
	D – around wound
3 – Clear or haemoserous discharge	A – at one point only (< 2 cm)
	B – along wound (> 2 cm)
	C – large volume
	D – prolonged (> 3 days)
4 – Pus/purulent discharge	A – at one point only (< 2 cm)
	B – along wound (> 2 cm)
5 – Deep or severe wound infection with or without tissue breakdown	

### Study population

Patients with the following criteria are eligible for inclusion: resection of primary or recurrent rectal cancer by APR, age of 18 years or older, ability to complete follow-up, and written informed consent. Exclusion criteria are total pelvic exenteration or sacral resection above level S4/S5, resection by an intersphincteric APR, biological mesh closure of the pelvic floor, severe systemic diseases affecting wound healing except for diabetes (i.e. renal failure requiring dialysis, liver cirrhosis, and immune compromised status like HIV), collagen disorders (i.e. Marfan syndrome, Ehler-Danlos syndrome) and enrolment in other trials with the same primary endpoint.

### Treatment strategies

In both study arms, APR can start with either the abdominal or the perineal phase. The abdominal phase of the APR can be performed via either laparoscopic or open surgery. The extent of perineal skin excision will be as limited as oncologically justified. The perineal phase of the APR will be performed according to the principles of a complete or limited extralevator APR. This means that the levator muscles will be transected laterally on both sides in order to leave a muscular cuff all around the resection specimen, or that part of the levator muscles will be resected only at the side of the tumour. Routine removal of the coccyx will not take place, unless based on oncological principles or needed to improve surgical visibility. An omentoplasty will not routinely be performed. A transabdominal drain will be placed and removed after 4 days or when the drain production is beneath 100 cc/24 h. The APR specimens will be classified according to Phil Quirke’s classification [6]. Postoperatively, the Enhanced Recovery After Surgery (ERAS) protocol will be followed and the sutures will be removed after 14 days at the outpatient clinic.

#### Perineal closure control arm

Standard practice in the Netherlands and the participating centre in the UK is primary closure of the perineum. This consists of stitching the perineal ischioanal fat together using interrupted 2.0 Vicryl sutures. Afterwards, the subcutaneous fat will be closed using interrupted 2.0 Vicryl sutures. A Redon drain (CH10) will standardly be placed between these layers. Subsequently, the skin will be closed using interrupted Vicryl 3.0 sutures. The perineal redon suction drain will be removed after 14 days or when the production is less than 30 cc/24 h.

#### Perineal closure experimental arm

Perineal closure will be performed or supervised by a surgeon experienced with the gluteal turnover flap. The first gluteal turnover flap procedure at a local participating hospital will always be supervised by an experienced (plastic) surgeon from the study group. When experience with the flap is not sufficiently present after the first time, supervision will be continued as long as required. The patient will be positioned either in prone or lithotomy position as preferred by the operating surgeon. The flap is marked on the gluteal skin on either the right or the left side of the surgical defect with a maximum width of about 2.5 cm. The half-moon shaped skin island is deepithelialised. Subsequently, the flap is developed by incising the dermis and subcutaneous fat with approximately a 45 degree level towards lateral. Gluteal perforators are not selectively dissected, and for this reason there is no need for pre-operative Doppler identification of perforators, which simplifies the procedure. Once developed, the subcutaneous flap is placed into the perineal defect and the deepithelialised dermis is fixed to the contralateral pelvic floor remnant with Vicryl 2.0 sutures. Afterwards, the perineum is closed in layers over the gluteal flap using interrupted 2.0 Vicryl sutures for the subcutaneous fat and Vicryl 3.0 interrupted sutures for the skin. A Redon drain (CH10) will standardly be placed between the flap and the subcutaneous fat and removed after 14 days or when the production is less than 30 cc/24 h. The procedure with accompanying video has been published previously [7].

### Outcome parameters

#### Primary endpoint

The primary endpoint of the study is the percentage of uncomplicated perineal wound healing at 30 days postoperatively. Uncomplicated perineal wound healing is defined as a Southampton wound score of less than two.

#### Secondary endpoints

Secondary endpoints are perineal wound healing according to the Southampton wound grading at 14 days, 3, 6 and 12 months postoperatively; postoperative pain score, the effect of neo-adjuvant treatment on perineal wound healing, incidence of persistent perineal or presacral sinuses both clinically and by imaging (routine follow-up CT), need for re-intervention or re-admission related to perineal wound problems, incidence of symptomatic and asymptomatic perineal hernia during follow-up, length of hospital stay, and quality of life and urogenital function (EQ-5D, EORTC-30, EORTC-29, SF36, UDI-6, IIQ-7, IIEF, FSFI, FSDS-R).

### Sample size calculation

The proportions of patients with uncomplicated perineal wound healing between both study arms will be compared and analysed by the intention-to-treat approach. The hypothesis is that gluteal turnover flap closure increases the uncomplicated perineal wound healing rate compared to primary closure of the perineum.

Our recently conducted pilot study showed promising results of the gluteal turnover flap with a flap failure in zero of ten patients included, and no Clavien-Dindo complications of three or higher within 30 days of surgery. Among the ten patients, there were four minor complications and no major wound complications requiring re-intervention at 30 days postoperatively. The minor complications consisted of two perineal wound dehiscences, one perineal infection necessitating manual drainage and antibiotic therapy, and one non-infected perineal seroma that also required manual drainage. A recent published case series of 13 patients undergoing an APR for rectal cancer with a similar flap procedure showed no cases of flap loss and no donor site or major perineal morbidity [8]. Combining the outcome of our pilot study with the recently published case series results in an uncomplicated wound healing rate of 83% (19/23). In current literature an increase of uncomplicated perineal wound healing of approximately 20% is to be expected when a muscle flap is being used. However, these data are mostly derived from cohort series in which different kinds of flaps were being used, with different definitions of complicated perineal wound healing. Therefore the currently available literature is difficult to interpret with regard to perineal wound healing. In our previously conducted randomised controlled trial (the BIOPEX-study), there was an uncomplicated perineal wound healing percentage of 66% after primary perineal wound closure. Extrapolating the reported improvement in perineal wound healing, a total number of 146 patients (73 per group) are needed to be able to detect a 20% increase in primary perineal wound healing by use of a gluteal turnover flap (from 65 to 85%), applying a Chi-Square test with a two-sided 0.05 significance level and with 80% power. With an estimated drop-out of 10%, a total number of 160 patients are required (80 per group).

### Data analysis

#### Primary endpoint

An intention-to-treat analysis will be performed on the primary outcome using a two-sided Chi-square test to compare the two study groups. A *p-*value of < 0.05 is considered statistically significant. Specific wound complications will be compared between the groups as categorical variables using the Mann-Whitney U test. A Kaplan-Meier survival analysis will be used to analyse the differences in time to perineal wound healing between the two study groups. Statistical analyses will be performed using SPSS software for Windows version 26.

#### Secondary endpoints

Relative risk with 95% confidence interval will be used to analyse treatment effect according to intention-to-treat. Analysis of binary secondary outcome measures will be performed similar to the primary outcome (such as perineal hernia rate, re-intervention rate, wound complication etc.). All *p*-values will be two-tailed and a p-value of < 0.05 will be considered statistically significant. Subgroup analyses will employ a test of interaction to explore whether there is evidence that the treatment effects differ across subgroups. As with all subgroup analyses these will be interpreted with caution, and will be considered hypothesis generating.

#### Quality of life and urogenital function

Graphic representation will be used to portray quality of life and urogenital function data (EQ-5D, EORTC-30, EORTC-29, SF36, UDI-6, IIQ-7, IIEF, FSFI, FSDS-R) across all follow-up moments, analysed according to the manuals, and will presented as domain and summarised scores. Questionnaire outcome comparisons will be analysed using linear mixed models. All analyses will be according to the intention-to-treat principle.

### Data monitoring

An independent Data Safety Monitoring Board (DSMB) committee has been established to perform ongoing safety surveillance and interim analyses on the safety data. The DSMB is composed of two independent clinicians and one independent epidemiologist. None of the members has conflict of interest with the sponsor of the study. An interim analysis will be performed at 80 included patients (of the total of 160 patients). The steering committee will be supplied with the number of (serious) adverse events in both groups. If there is a skewed distribution of the number of (serious) adverse events between the two groups, an efficacy analysis can be performed at the discretion of the steering committee. Following these interim analyses, the steering committee will advise upon continuation of the trial.

### Ethics and safety

The study protocol (NL65461.018.18) has been approved by the medical ethical committee of the Academic Medical Centre, Amsterdam, the Netherlands. This study will be performed according to the principles of Good Clinical Practice.

## Discussion

A variety of techniques have been described for closure of the perineal defect following APR for rectal cancer. To date there is only one published randomised controlled trial on perineal wound closure after APR [2]. This study compared biological mesh closure with primary wound closure, and showed no improvement in primary wound healing after biological mesh closure. The absence of an effect is probably related to the properties of the mesh: a mesh adds strength to the pelvic floor, but does not fill the perineal defect. There is still a possibility of fluid accumulation in the perineal defect with the risk of secondary contamination.

Since it is thought that filling of the perineal defect is needed to prevent fluid formation, other perineal closure methods, such as autologous tissue flap closures are being increasingly used. A number of musculocutaneous transposition flaps and fasciocutaneous perforator flaps can be used for perineal closure after APR. The rectus abdominis muscle flap, gracilis flap, lateral thigh flap and the gluteal flap have all been described for this purpose [9–15]. The vertical rectus abdominis muscle (VRAM) flap is one of the flaps most often used for closure of relatively large perineal defects. It is well-vascularised, with sufficient vascular pedicle length and bulk, however it disturbs the abdominal wall integrity. In the era of laparoscopic rectal cancer surgery, a VRAM flap for routine perineal closure seems to be too invasive with relatively high donor site morbidity. Furthermore, failure rates as high as 15% have been reported [9, 13, 16].

The gracilis flap can provide good coverage for the anterior perineal wound. However, due to the distal positioning of the vascular pedicle the mobility is restricted, limiting its use for filling the perineal defect. According to the current literature, the lateral thigh flap is also a good alternative for the reconstruction of the perineum. It may prevent perineal herniation due to the strong tensor fasciae latae [9]. Although promising, the literature on this topic is scarce, especially in oncological patients. Besides that, the lateral thigh flap is most often performed by a plastic surgeon, has a difficult closure of the donor site, may require an additional skin graft and substantially increases surgery duration and patient morbidity.

Finally, a gluteal flap can be used for perineal closure after APR and can be performed as a perforator flap (IGAP/SGAP) or a myocutaneous flap [17]. The gluteal perforator flap lies outside the radiated field and transfers solely well-vascularised skin and subcutaneous tissue into the perineal wound. Due to the transfer of only skin and subcutaneous tissue, patients experience less pain and morbidity at the donor site compared to the gluteal myocutaneous flap [8, 18]. The gluteal myocutaneous flap has more bulk upon positioning in the pelvic wound, but due to the atrophy of the muscle the myocutaneous flap loses its size over time. Furthermore, the gluteal myocutaneous flap results in a big scar on the buttock with implications for daily life activities.

A recent meta-analysis of ten studies, including 8 studies using VRAM flap and 2 studies using gracilis flaps, found a significant reduction in perineal complications by flap reconstruction after APR compared to primary perineal wound closure (pooled perineal wound complication rate of 35% versus 52%, OR 2.17 (95% CI 1.49–3.15), heterogeneity I2 = 0%) [11]. Especially the major perineal wound complications were reduced (pooled proportions of 8% vs 25%, OR 3.64 (95% CI 1.70–7.79), I2 = 0%). The drawback of the current flap series is that they often lack control, and often describe selective populations with larger defects which makes comparison difficult. Moreover, no randomised controlled trials comparing muscle flaps to other techniques have been published until now. Currently, there is one other open trial (trial registration number: NCT01347697), which started approximately 5 years ago, and randomises between gluteus maximus myocutaneous flap and acellular porcine collagen implant [19].

The perineal dead space after APR is often relatively limited and does not routinely require a large bulky tissue flap. A flap should therefore be relatively simple to perform, requiring only limited additional operative time, should be able to be combined with laparoscopic surgery and performed in both prone and supine positions, and should not add additional scars. The gluteal turnover flap, which constitutes the experimental intervention in the present trial, is a modified perforator flap which fulfils these criteria [8]. The gluteal turnover flap seems to be a promising method for the purpose of routine perineal closure after APR for primary and recurrent rectal cancer that does not require exenterative procedures. In addition to its positive effect on the perineal wound healing, it is expected that perineal hernia incidence will be reduced, by anchoring the strong dermis onto the contralateral remnant of the levator ani.

The use of neo-adjuvant therapy in the Netherlands has been reduced in the last couple of years. Therefore, non-radiated patient are also considered eligible in the BIOPEX-2, while only radiated patients were included in the BIOPEX trial. Neo-adjuvant therapy is one of the most import risk factors for perineal wound complications, and stratification will be used to balance the study arms for this factor.

It was decided to use the same control intervention in the BIOPEX-2 trial as used in the BIOPEX trial, namely primary perineal closure. The main reason was because no superiority of biomesh closure on the primary endpoint could be demonstrated. Although a significant difference in perineal hernia rate was found for the experimental arm in the BIOPEX study as a secondary finding, biological mesh closure did not become the standard of care in the Netherlands. The relatively high costs and relatively small absolute difference in perineal hernia rate were the main reasons for continuing primary closure as routine daily care in most Dutch institutions. Furthermore, the reduced perineal hernia rate needs further confirmation by long-term follow-up.

In conclusion, tissue flaps used to fill perineal dead space have been suggested to improve perineal wound healing, but currently used flaps are relatively invasive and have several drawbacks. The gluteal turnover flap seems to be a promising method, which is less invasive, does not add donor site scarring, and is relatively easy to perform. High quality evidence on its efficacy is needed, for which reason the BIOPEX-2 has been designed.

## Data Availability

The datasets used and/or analysed during the current study will be available from the corresponding author on reasonable request.

## Abbreviations

BIOPEX-2 study: Title of this study, which refers to the previous trial on this subject (BIOlogical mesh closure of the Pelvic floor after EXtralevator abdominoperineal resection for rectal cancer)
APR: Abdominoperineal resection
TME: Total mesorectal excision
S4/S5: 4th / 5th sacral vertebra
VRAM: Vertical rectus abdominis muscle
EQ 5D-5 L (Euroqol): This questionnaire is a simple, generic instrument for describing and valuing health related quality of life. It includes 5 items (mobility, personal care, daily activities, pain, and anxiety-depression) that are answered on a 3-point scale ranging from no problems (level 1) to extreme problems (level 3). The health valuations are used together with the times spent in those health states to derive quality adjusted life years (QALY).
Global quality of life (EORTC-QLQ-C30-QL2): This sub-questionnaire contains the 2 items of the global quality of life dimension of the EORTC-QLQ-C30 questionnaire.
Global quality of life (EORTC-QLQ-CR29): This questionnaire is developed to assess the quality of life in colorectal patients.
Quality of Life questionnaire (SF36): This questionnaire measures the relationship between a client’s quality of life and other behaviours or afflictions.
UDI-6 and IIQ-7: The Urogenital Distress Inventory (UDI-6) and Incontinence Impact Questionnaire (IIQ-7) assess symptom distress and the impact on daily life of urinary incontinence.
IIEF: The international index of erectile function assesses erectile dysfunction.
FSFI: The Female Sexual Functioning Index assesses and helps monitor sexual function and level of dysfunction in women.
FSDS-R: The Female Sexual Distress Scale-Revised is a screening questionnaire for measuring sexually related personal distress in women with female sexual dysfunction.
DSMB: Data Safety Monitoring Board
